# GIANT CELL TUMOR OF THE DISTAL RADIUS: FACTORS ASSOCIATED WITH LOCAL RECURRENCE

**DOI:** 10.1590/1413-785220253301e289573

**Published:** 2025-02-03

**Authors:** William Bernardo Specht Rabuske, Michelle Ghert, Bruno Pereira Antunes, Carlos Roberto Galia, Julie Francine Cerutti Santos Pestilho, Gabriella Sityá Moojen da Silveira, Eduardo Areas Toller, Olavo Pires de Camargo, Edgard Eduard Engel, Suely Akiko Nakagawa, Alex Guedes, Ricardo Gehrke Becker

**Affiliations:** 1 Hospital de Clínicas de Porto Alegre (HCPA), Porto Alegre, RS, Brazil; 2 University of Maryland, Baltimore, MD, USA; 3 McMaster University, Hamilton, Canada; 4 Instituto do Câncer Infantil, Porto Alegre, RS, Brazil; 5 Hospital de Amor, Barretos, SP, Brazil; 6 Universidade de Sao Paulo, Faculdade de Medicina, Hospital das Clinicas HC-FMUSP, Sao Paulo, SP, Brazil; 7 Faculdade de Medicina de Ribeirão Preto. Universidade de São Paulo ou Ribeirão Preto Medical School. University of São Paulo; 8 Hospital A. C. Camargo Cancer Center, São Paulo, SP, Brazil; 9 Hospital Santa Izabel, Salvador, BA, Brazil

**Keywords:** Bone Neoplasms, Giant Cell Tumors, Giant Cell Tumor of Bone, Curettage, Denosumab, Recurrence, Neoplasias Ósseas, Tumores de Células Gigantes, Tumor de Células Gigantes do Osso, Curetagem, Denosumab, Recidiva

## Abstract

**Objectives:**

To assess patient and tumor characteristics and treatment outcomes, focusing on local recurrence rates based on treatment type.

**Methods:**

This is a retrospective review of cases of GCTB of the distal radius, identified from the databases of 74 patients in Brazilian institutions specializing in musculoskeletal tumor treatment. Data were collected from electronic and paper medical records by 18 centers between 1989 and 2021. Variables included demographic data, clinical presentation, treatment-related factors, and primary outcome (local recurrence rate).

**Results:**

Among the 74 patients in the study, the mean age at diagnosis was 32.6 years, with a slight female predominance. Pathological fractures on presentation were observed in 15.7% of patients, and pulmonary metastasis in 1.4%. Treatment approaches were divided equally between intralesional curettage and en bloc resection. The overall local recurrence rate was 25.7% and was higher in patients treated with intralesional curettage (35.1%) compared to resection (16.2%).

**Conclusions:**

The study confirms high recurrence risk with intralesional curettage, emphasizing the need for standardized protocols and improved surgical techniques to reduce recurrence rates and enhance outcomes for distal radius GCTB patients. ***Level of Evidence III; Retrospective Cohort Study.***

## Resumo

**Objetivos:**

Avaliar as características dos pacientes e dos tumores, e os resultados do tratamento, focando nas taxas de recorrência local baseadas no tipo de tratamento.

**Métodos:**

Relata-se uma revisão retrospectiva de casos de TCG do rádio distal, identificados a partir dos bancos de dados de 74 pacientes tratados em instituições brasileiras especializadas em tratamento de tumores musculoesqueléticos. Os dados foram coletados de registros médicos eletrônicos e físicos por 18 centros entre 1989 e 2021. As variáveis incluíram dados demográficos, apresentação clínica, fatores relacionados ao tratamento e desfecho primário (taxa de recorrência local).

**Resultados:**

Dos 74 pacientes incluídos no estudo, a idade média no diagnóstico foi de 32,6 anos, com uma leve predominância feminina. Fraturas patológicas na apresentação foram observadas em 15,7% dos pacientes, e metástase pulmonar em 1,4%. As abordagens de tratamento foram divididas igualmente entre curetagem intralesional e ressecção em bloco. A taxa geral de recorrência local foi de 25,7%, sendo maior em pacientes tratados com curetagem (35,1%) em comparação com a ressecção (16,2%).

**Conclusões:**

O estudo confirma o alto risco de recidiva com uso da curetagem, enfatizando a necessidade de protocolos padronizados e técnicas cirúrgicas aprimoradas para reduzir as taxas de recorrência e melhorar os resultados para pacientes com TCG do rádio distal. **Nível de Evidência III; Estudo de Coorte retrospectivo.**

## INTRODUCTION

Giant cell tumor of bone (GCTB) is a primary benign yet aggressive bone lesion, representing approximately 5% of all primary bone tumors in Western countries. It is slightly more common in women, with a peak incidence between the ages of 20 and 50. These tumors frequently occur in the epiphysis of long bones, with a preference for the knee region and the distal radius.^1^,^2^,^3^ Clinically, patients present with pain, swelling, and occasionally pathological fractures. Although metastatic disease is infrequent, occurring in 1 to 5% of cases, some authors suggest that the distal radius presents a higher risk.^4^ Death due to GCTB is very rare, with the greatest tumor morbidity related to the function of the affected bone and joint.^5^

*Campanacci’*s classification has been used to determine the aggressiveness of GCTB based on x-ray images. Grade 1 lesions are confined to the bone, grade 2 lesions show some expansion of the cortex, and grade 3 lesions break through the cortex with soft tissue involvement.^6^ Management of GCTB typically involves surgery, with intralesional curettage being the preferred approach for grade 1 and 2 lesions, while resection is recommended for grade 3 lesions due to their more aggressive behavior and lack of a contained defect. However, the reported local recurrence rate for distal radius tumors is high, ranging between 25% and 50% depending on the surgical approach, tumor extent, and radiographic grade.^4^,^7^,^8^

**Figure 1:**
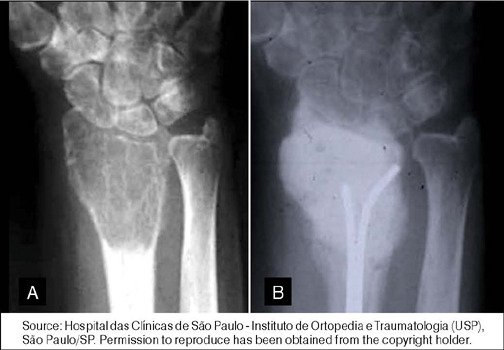
(A) X-ray of the wrist showing a Campanacci grade 3 giant cell tumor of bone (GCTB) of the distal radius; (B) The patient was treated with intralesional curettage, adjuvants, and cement filling.

Source: Hospital das Clínicas de São Paulo - Instituto de Ortopedia e Traumatologia (USP), São Paulo/SP. Permission to reproduce has been obtained from the copyright holder.

**Figure 2:**
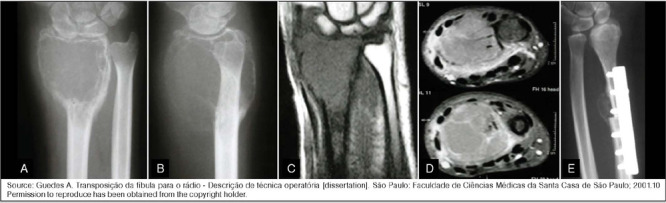
(a) X-ray (front view) and (b) X-ray (lateral view) of the wrist illustrating a Campanacci grade 3 giant cell tumor of bone (GCTB) of the distal radius; (c) magnetic resonance imaging (MRI) T1 coronal and (d) MRI T2 axial images; (e) The tumor was treated with resection and reconstruction using a fibular autologous bone graft.

Source: Guedes A. Transposição da fíbula para o rádio - Descrição de técnica operatória [dissertation]. São Paulo: Faculdade de Ciências Médicas da Santa Casa de São Paulo; 2001.10 Permission to reproduce has been obtained from the copyright holder.

**Figure 3:**
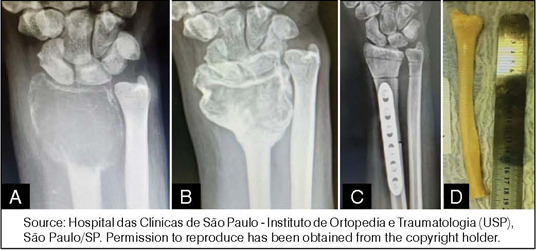
(a) Pre-treatment and (b) post-treatment X-rays of a distal radius giant cell tumor of bone (GCTB) treated with denosumab; (c) X-ray after resection and reconstruction using (d) an allograft specimen.

Source: Hospital das Clínicas de São Paulo - Instituto de Ortopedia e Traumatologia (USP), São Paulo/SP. Permission to reproduce has been obtained from the copyright holder.

The choice between intralesional curettage (Figure 1) and resection (Figures 2 and 3) depends on the severity of the lesion and patient characteristics.^7^,^9^ Intralesional curettage is often associated with lower surgical morbidity and preservation of limb function because it preserves the joint surface, but has a higher recurrence rate, especially in grade 3 lesions. On the other hand, resection is more aggressive, resulting in better oncologic control but significant functional loss, particularly in large tumors.

In this study, we reviewed a multicenter cohort of patients treated for distal radius GCTB in national tumor centers in Brazil. The aim of the study was to assess patient and tumor characteristics and to describe the treatment outcomes of GCTB located in the distal radius in the context of an emerging economy.

## MATERIALS AND METHODS

This study is a retrospective review of 74 cases of GCTB of the distal radius, identified from the databases of 643 patients with GCTB from various Brazilian institutions specializing in musculoskeletal tumor treatment. The study received ethical approval from Hospital de Clínicas de Porto Alegre (HCPA) and all participating institutions (REB 94280918.0.0000.5327). All procedures were conducted in accordance with the ethical standards of Resolution 466/2012 of the Brazilian Ministry of Health’s National Health Council and the Declaration of Helsinki. Informed consent was waived because of the retrospective nature of the study.

Data were collected from electronic and paper medical records by 18 participating centers between 1989 and 2021. To ensure participant confidentiality, each individual was assigned a numeric code. Data were transmitted to the coordinating center via an encrypted email system. Upon receipt, the data were thoroughly examined to resolve any discrepancies or inconsistencies. Cases with conflicting variables were returned to their respective centers for clarification and then re-examined by the coordinating center. The collected data were stored in MS Excel and SPSS version 28.0 software programs.

The extracted variables were categorized into: demographic variables (gender, age, region of the country where the patient received treatment), clinical presentation variables (pulmonary metastasis, pathological fracture, and *Campanacci* classification based on radiographic appearance), treatment-related variables (type of surgery – intralesional curettage, resection - type of filling after curettage - cement, bone graft -, surgical adjuvants used -drilling, alcohol, ablation - and use of denosumab), and primary outcome (local recurrence rate).

Inclusion criteria were: (1) histopathological diagnosis of GCTB of the distal radius; (2) treatment of the primary tumor performed at one of the participating centers; (3) availability of patient medical records for analysis by the coordinating center. A total of 74 patients met the inclusion criteria. Collaborative efforts between the participating entities identified and corrected data discrepancies and gaps. However, among the 74 patients evaluated, instances of missing information were observed in 3 patients for pulmonary metastases, 4 patients for pathological fractures, and 2 patients for cavity filling type. These data deficiencies were predominantly due to the loss of historical medical records and inconsistencies in documentation procedures among the various participating institutions.

The primary outcome examined was the local recurrence rate, which was reviewed according to the type of surgery, the use of denosumab before intralesional curettage, the number of adjuvants used during surgery, and tumor aggressiveness according to the *Campanacci* classification.^6^

## RESULTS

### Patient and Treatment Characteristics

Table 1. In this analysis of 74 patients with GCTB of the distal radius, the mean age at diagnosis was 32.6 years. Regarding sex distribution, 43 patients (58.1%) were female, while 31 patients (41.9%) were male. Geographically, 23 patients (31.1%) were from the South region, 10 patients (13.5%) from the Northeast, 40 patients (54.1%) from the Southeast, and 1 patient (1.4%) from the North. In terms of *Campanacci* classification, 25 patients (33.8%) had tumors classified as *Campanacci* 1 or 2, while 49 patients (66.2%) had *Campanacci* 3 tumors. Pathological fracture was observed on presentation in 11 patients (15.7%). Only 1 patient (1.4%) presented with pulmonary metastasis. Denosumab was used in 13 (17.6%) patients, 11 for an effort to reduce tumor size, and 2 for local recurrence.

**Table 1:** Patient and treatment characteristics.

Variables	Total Sample (n=74)
Age at diagnosis (years)	Mean ± SD: 32.6 ± 11.5
**Sex – n (%)**	
Female	43 (58.1)
Male	31 (41.9)
**Campanacci classification – n (%)**	
I/II	25 (33.8)
III	49 (66.2)
**Patients per region in Brazil – n (%)**	
South	23 (31.1)
Northeast	10 (13.5)
Southeast	40 (54.1)
North	1 (1.4)
Pulmonary Metastasis – n (%)	1 (1.4)
Pathological Fracture – n (%)	11 (15.7)
**Type of Surgery – n (%)**	
Intralesional curettage	37 (50.0)
Resection	37 (50.0)
Type of Filling – n (%)*	
Cement	29 (80.6)
Cement + Graft	2 (5.6)
Bone Graft	5 (13.9)
Adjuvants – n (%)*	
None	7 (9.4)
Single	14 (18.9)
Combined	16 (21.6)
Types of Adjuvants – n (%)*	
Drilling	17 (45.9)
Alcohol	10 (27.0)
Fulguration	24 (64.9)
Local Recurrence – n (%)	19 (25.7)
Patients treated with Intralesional curettage	13 (35.1)
Patients treated with resection	6 (16.2)
Denosumab – n (%)	13 (17.6)

* Intralesional curettage only (n=37).

Intralesional curettage was performed on 37 patients and resection on 37 patients. Among the patients who underwent curettage, 7 patients (18.9%) did not receive a surgical adjuvant, 14 patients (37.8%) received a single surgical adjuvant, and 16 patients (43.2%) received combined surgical adjuvants. Specifically, 17 patients (45.9%) underwent adjuvant treatment with high-speed burr, 10 patients (27.0%) received alcohol or phenol, and 24 patients (64.9%) underwent ablation. For cavity filling, 29 patients (78.4%) had reconstruction with cement, 2 patients (5.4%) with cement and bone graft, and 5 patients (13.5%) with bone graft.

### Local Recurrence

Table 2. The local recurrence rate was 25.7% (19 patients). When analyzed by type of surgery, the local recurrence rate for patients who underwent intralesional curettage was 35.1% (13 patients), while for those who underwent resection it was 16.2% (6 patients). According to *Campanacci* classification, the local recurrence rate was 28.5% for grade 3 and 20% for grades 1 and 2. Local recurrence occurred in 13.3% of patients with pathological fractures, compared to 86.7% in those without. One patient who presented with pulmonary metastasis also developed local recurrence.

**Table 2:** Local recurrence.

Variables	Recurrence (n=19)	No Recurrence (n=55)
**Sex – n (%)**		
Female	12 (63.2)	31 (56.4)
Male	7 (36.8)	24 (43.6)
Age at diagnosis (years) – median	32.2 ± 9.1	33.5 ± 12.3
**Campanacci grade – n (%)**		
I/II	5 (26.3)	20 (36.4)
III	14 (73.7)	35 (63.6)
Pulmonary Metastasis – n (%)	1 (5.3)	0 (0.0)
Pathological Fracture – n (%)	2 (8.7)	13 (14.4)
**Type of Surgery – n (%)**		
Intralesional curettage	13 (68.4)	24 (43.6)
En bloc resection	6 (31.6)	31 (56.4)
Type of Filling – n (%)*		
Cement	8 (61.5)	21 (87.5)
Cement + Graft	0 (0.0)	1 (4.2)
Bone Graft	4 (30.8)	1 (4.2)
None	1 (7.7)	1 (4.2)
Number of Adjuvants – n (%)*		
None	3 (23.1)	5 (20.8)
Single	5 (38.5)	9 (37.5)
Combined	5 (38.5)	10 (41.7)

* Intralesional curettage only (n=37).

Regarding sex, 63.2% of patients with recurrence were female, while 36.8% were male. The mean age at diagnosis for patients with recurrence was 32.2 years, while for patients without recurrence it was 33.5 years. Among patients who were treated with denosumab, 23.1% had recurrence, compared to 26.2% of patients who were not treated with denosumab. Patients treated with denosumab and intralesional curettage had a local recurrence rate of 15.3% (2/13), compared to 20% (1/5) of those treated with denosumab and resection.

Patients who did not receive any surgical adjuvants after intralesional curettage had a local recurrence rate of 37.5%, while those who received single or combined surgical adjuvant had rates of 35.7% and 33%, respectively. In terms of cavity filling after curettage, 30.8% of patients with recurrence were reconstructed with bone graft, while 61.5% were reconstructed with cement.

## DISCUSSION

The study reported on a multicenter retrospective cohort of 74 patients with GCTB of the distal radius, with a mean age of 32.6 years and a slightly higher percentage of females. Geographically, most patients were from the Southeast region of Brazil. Clinical features included a notable occurrence of pathological fractures at presentation and only one patient presenting with pulmonary metastasis. Treatment approaches were divided between intralesional curettage and resection, with varying use of adjuvant therapies such as denosumab. The study identified a considerably high rate of local recurrence of 25.7%, particularly in patients treated with curettage, highlighting the challenges of managing this aggressive benign bone tumor in this anatomic location.

The findings of this study align with existing literature on the management of GCTB of the distal radius. Pazionis et al. conducted a systematic review comparing resection and intralesional curettage. Their results indicated a higher recurrence rate for curettage (31%) compared to wide excision (8%).^7^ Similarly, our study found a 35.1% recurrence rate for curettage versus 16.2% for resection. These consistent findings underscore the challenges of managing GCTB in the distal radius, where preserving function must be balanced against the risk of recurrence.^7^

Montgomery et al. emphasized the aggressive nature of GCTB and the preference for surgical management, often supplemented with adjuvant therapies to reduce recurrence.^11^ However, this and other studies have reported lower overall recurrence rates than those reported herein. The higher local recurrence rate in our series may be due to the higher-than-expected percentage of patients with *Campanacci* grade 3 lesions (66.2%). Patients with grade 3 tumors tend to exhibit higher rates of local recurrence, especially after intralesional curettage.^4^,^8^,^12^

Differences in recurrence rates could also be attributed to the lack of access to advanced imaging, and the prolonged waiting times for access to a referral center, which may not have been uniformly available across the centers in our study. In their series, Wysocki et al. noted that centers with access to high-quality imaging and surgical tools tend to report better outcomes in patients with GCTB of the distal radius.^13^ Similarly, treatment delays can impact both functional outcomes and local recurrence rates. This disparity underscores the critical need for standardized treatment protocols and prompt access to specialized care to enhance patient outcomes in Brazil. It is likely that meticulous surgical techniques and/or the use of adjuvant therapies may reduce local recurrence rates. The use of adjuvants after intralesional curettage in our series did not appear to reduce the rate of local recurrence. In fact, Pazionis et al. and other reviews indicate that recurrence rates can be significantly reduced with careful surgical planning with or without the use of adjuvants.^7^ This highlights the potential of our study to inform future treatment guidelines and improve outcomes for patients with distal radius GCTB.^7^,^14^,^15^

The study has several limitations. Data collection spanned over three decades, during which surgical techniques and adjuvant therapies evolved, potentially introducing variability in treatment outcomes. Additionally, missing data in some variables could have affected the analysis. Finally, selection bias will have played a major role in determining surgical approach, further qualifying our conclusions. Despite these limitations, the study’s strengths include its multicenter design and the relatively large sample size for a rare tumor, providing a comprehensive overview of GCTB management in Brazil.

## CONCLUSION

This study highlights the challenges and outcomes associated with treating GCTB of the distal radius in Brazil. The findings underscore the high recurrence rates in patients with distal radius GCTB, particularly when treated with intralesional curettage compared to resection. There was a high prevalence of cases with more aggressive tumors (*Campanacci* grade 3), which likely resulted in higher local recurrence rates. The use of combined or single adjuvants did not reduce recurrence rates in this series of GCTB of the distal radius.
